# Am I Overweight? A Longitudinal Study on Parental and Peers Weight-Related Perceptions on Dietary Behaviors and Weight Status Among Adolescents

**DOI:** 10.3389/fpsyg.2016.00083

**Published:** 2016-02-04

**Authors:** Karolina Zarychta, Barbara Mullan, Aleksandra Luszczynska

**Affiliations:** ^1^SWPS University of Social Sciences and HumanitiesWroclaw, Poland; ^2^Curtin University, BentleyWA, Australia; ^3^University of Colorado at Colorado Springs, Colorado SpringsCO, USA

**Keywords:** nutrition behavior, weight status, perceptions of weight status, adolescence, social influence

## Abstract

**Objective:** An investigation of the interplay between various types of adolescents’ perceptions of weight status in predicting adolescents’ nutrition behavior and their body mass was conducted. In particular, it was hypothesized that the relationship between parental and peers’ perceptions of their own weight status (reported by adolescents) and objectively measured weight status of adolescents would be mediated by three types of adolescents’ weight status perceptions (adolescents’ own weight perceptions, parental perceptions of adolescents’ weight status perceived by participants, and peers’ perceptions of adolescents’ weight status perceived by participants) and by adolescents’ nutrition behaviors.

**Design:** Data were collected twice, with a 13-month follow-up. Participants (*N* = 1096) were aged 14–20, with BMI ranging from 16.20 to 41.21. Multiple mediation analysis with two sequential mediators was applied.

**Main outcome measures:** At the baseline adolescents completed the questionnaire assessing their nutrition behaviors and weight status perceptions. Weight and height were measured objectively at baseline and follow-up.

**Results:** Two types of weight perceptions (adolescents’ own weight status perceptions, peers’ perceptions of adolescents’ weight status reported by participants), and adolescents’ nutrition behaviors mediated the relationship between the others’ own weight perceptions and adolescents’ weight status. No indirect effects of others’ own weight perceptions on adolescents’ weight status through parental perceptions were found.

**Conclusion:** Adolescents’ nutrition behaviors and body weight status depend on what they think about their own weight status and what they think of their peers’ perceptions, but do not depend on what adolescents think of their parents’ perceptions.

## Introduction

Overweight and obesity in adolescents has been increasing over the last 30 years (National Center for Health Statistics, 2013; Ogden et al., 2014). Internationally, 13.4 to 21.7% of preadolescents and adolescents are overweight or obese (Currie, 2012) when applying the International Obesity Task Force threshold for excessive weight (Cole et al., 2000). According to the World Health Organization (World Health Organization [WHO], 2012) adolescents should eat at least four healthy meals a day (including fresh fruit and vegetables) to maintain optimal body weight. The causal link between nutrition behavior and increased body mass index (BMI) is well established (Piernas and Popkin, 2010). Adolescents with excessive weight may make attempts to stick to a healthy nutrition in order to regulate their body mass (cf. Szczepanska et al., 2013). These attempts, however, are often unsuccessful (Robertson et al., 2014).

Perceptions of weight status are one of the key cognitive factors emphasized as significant determinants of both healthy nutrition behaviors and BMI (Chen et al., 2014; Perkins et al., 2014; Tompkins et al., 2014). Research has indicated that it is not the objective reality (adolescents and others’ actual weight status) but the subjective perception of that reality (adolescents and others’ perceived weight status) that is the strongest correlate of healthy eating behavior and BMI (Perkins et al., 2014).

Social environment including the micro-environment (e.g., schools, home, and neighborhoods) and its perceptions may help to explain changes in BMI among adolescents (Cislak et al., 2012). Theories explaining maintenance of health-related outcomes such as social cognitive theory (Bandura, 1969), emphasize the direct impact of social environmental variables on BMI and healthy behaviors, such as healthy nutrition behaviors (Kremers et al., 2006). Research on overweight and obesity in adolescence confirmed that the factors affecting the formation and maintenance of excessive body weight are perceptions others hold regarding their own overweight (Warschburger and Kroller, 2012). Parental perceptions of their own weight status are important factors in successful prevention and treatment of adolescents’ overweight and obesity (Keller et al., 2013). Parents who perceive themselves to be heavier were more concerned about adolescents’ weight (Keller et al., 2013). Moreover, research has indicated that peers’ perceptions of their own weight status may also affect adolescents’ weight status. Adolescents whose peers perceived themselves to be heavier and attempt to lose weight were more likely to change their nutrition behaviors compared to adolescents whose peers perceived themselves to be a healthy weight or were not attempting to lose weight (Perkins et al., 2010, 2014). In sum, a line of research suggested direct associations between parental and peers’ perceptions of their own weight status and BMI of adolescents. However, is likely that this connection is indirect rather than direct. Our study aims to investigate if the associations between parental and peers’ perceptions of their own weight status and adolescents’ BMI may be indirect and mediated by adolescents’ thoughts about their own weight status, others’ perceptions of adolescents’ weight status and nutrition behaviors.

Perceptions of weight status may be divided into self-perceptions or perceptions of others. Previous research has explored *adolescents’ perceptions of their own weight status* (what an individual think about their own weight; Rudolph et al., 2010; Ojala et al., 2012; Hisar and Toruner, 2013) and *others’ perceptions of their own weight status* (what an individual’s parents or peers think of the individual’s weight; Keller et al., 2013). Moreover, research has investigated *others’ perceptions of adolescents’ weight status* (what an individual’s parents or peers think of the individual’s weight; Chen et al., 2014; Sand et al., 2014; Tompkins et al., 2014) and individual *adolescents’ perceptions of others’ weight status* (what adolescents think about their parents or peers’ weight; Race Mackey and La Greca, 2008; Epuru et al., 2013).

Adolescents’ thoughts about their own weight status may be explained by how their peers and parents perceive their own weight. Research pointed out to an indirect link between peers/parental perceptions of their own overweight and adolescents’ body weight, with adolescents’ perceptions of their weight status playing the mediating role. For example, others’ perceptions of their own overweight status predicted adolescents thinking more often about themselves as overweight/obese (Perkins et al., 2014). Attending school with overweight or obese peers may affect not only adolescents’ perceptions of own weight status (Maximova et al., 2008; Fredrickson et al., 2014), but it may also influence adolescents’ perceptions of peer weight norms, leading to over- or underestimation of their weight status (Perkins et al., 2014). In general, various weight status perceptions are consider one of the most important motivating factors for nutrition behaviors (Hisar and Toruner, 2013). Therefore, it may be assumed that the effects of peers/parental perceptions of their own overweight on adolescents’ weight status are indirect. The two potential sequential mediators are adolescents’ perceptions of their weight status (their own, as seen by their peers and parents) and nutrition behavior, respectively.

The majority of research on adolescents’ thoughts about their own weight status (judged by themselves, their parents or peers) focused on the role of one type of thoughts, namely adolescents’ perceptions of their own weight status (i.e., judged by themselves). For example, studies indicated that higher weight status perceptions among adolescents are connected with a higher likelihood of engaging in weight-loss behaviors (such as healthy nutrition) and, in turn, with lower adolescents’ body mass (cf. Perkins et al., 2010; Chen et al., 2014; Tompkins et al., 2014). Thus, the perceptions of adolescents’ own weight status are amongst the strongest correlates of increased BMI (Ojala et al., 2012). The first steps in initiating change of eating behaviors and subsequent effective reduction of obesity/overweight include not only recognition of one’s actual overweight, but also perceptions of being overweight (Ojala et al., 2012; Rietmeijer-Mentink et al., 2013). Adolescents who can correctly recognize their objectively excessive weight (Yang et al., 2014) or subjectively perceive their weight as excessive (Bodde et al., 2014) were found to be more likely to attempt weight loss through changing their nutrition behaviors. In sum, adolescents’ perceptions of their own weight status are associated with adolescents’ body weight changes but the relationships may be indirect, with nutrition behaviors playing the mediating roles.

There is no sufficient or unequivocal empirical evidence which clarifies whether the relationship between others’ social influence variables (such as parental and peers’ perception of their weight status) and adolescents’ BMI is mediated by adolescents’ thoughts about their own weight status (judged by themselves, their parents or peers). The existing evidence provides some insight into the predictors of adolescents’ nutrition and weight status, indicating modeling (Kremers et al., 2006; Erkelenz et al., 2014), adolescents’ perceptions of their own weight status (Hisar and Toruner, 2013; Chen et al., 2014) and others’ perceptions of adolescents’ weight status perceived by participants (Perkins et al., 2014) as significant factors. However, the interplay between these factors or their synergistic effects on weight and behavior have not been studied.

So far, almost all studies have applied cross-sectional designs and focused primarily on direct and bivariate associations between these perceptions on the one hand and body weight or weight-related behaviors on the other hand. We found no studies testing the interplay between these three types of perceptions (i.e., adolescents’ perceptions of their own overweight status, others’ perceptions of their own overweight status and others’ perceptions of adolescents’ overweight status perceived by participants) and their mutual associations. Furthermore, although various types of adolescents’ perceptions of weight status has been studied extensively and have been found to be significant as far as adolescents’ healthy nutrition behaviors are considered (Hutchinson and Rapee, 2007; Helfert and Warschburger, 2013; Perkins et al., 2014), their long-term effects were not investigated thoroughly. Long-term effects of cognitions or perceptions and nutrition behavior (i.e., observed at follow-ups lasting at least 1 year) are crucial for predicting successful maintenance of healthy body weight (Cislak et al., 2012). Therefore, we tested whether weight perceptions and nutrition behavior would predict adolescents’ body weight at 13-month follow up, after controlling for baseline body weight.

**FIGURE 1:**
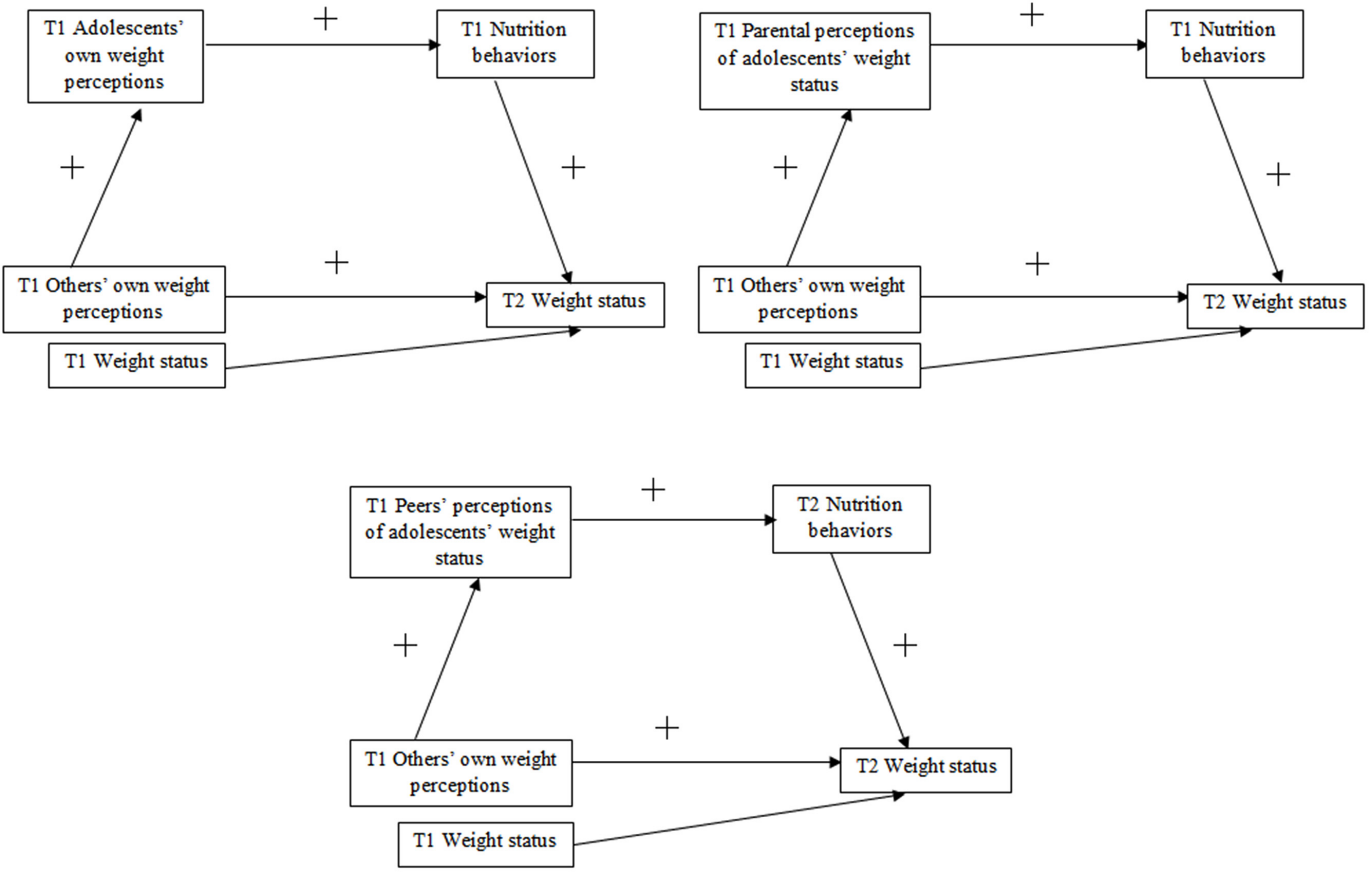
**Hypothesized associations between the others’ own weight perceptions, three types of weight status perceptions, adolescents’ nutrition behaviors and weight status.** T1 = Time 1, baseline; T2 = Time 2, 13-month follow-up.

Exploring the longitudinal associations and interplay between different types of weight status-related perceptions is of key importance in determining main, direct and indirect predictors of nutrition behaviors and, subsequently, body weight in adolescence. Thus, the present longitudinal study investigate the associations between adolescents’ subjective cognitions of parental and peers’ perceptions of their own weight status (individuals’ perception of what their parents and peers think of their own weight status; for ease of understanding “others’ own weight perceptions” will be used) and adolescents’ weight status, in the context of the potential mediators: three types of weight status perceptions: (1) adolescents’ own weight status perceptions (what an individual think about their own weight; for ease of understanding “adolescents’ own weight perceptions” will be used), (2) parental perceptions of adolescents’ weight status perceived by participants (individuals’ perception of what their parents think of the individuals’ weight; “parental perceptions”), or (3) peers’ perceptions of adolescents’ weight status perceived by participants (individuals’ perception of what their peers think of the individuals’ weight; “peers’ perceptions”), and adolescents’ healthy nutrition behaviors. We aimed at examining if the weight status perceptions and healthy nutrition behaviors, measured at the baseline, would explain adolescents’ weight status at long-term, 13-month follow-up (see **Figure 1**). The recognition of one’s actual weight or perceptions of being overweight are the first steps in initiating change in weight-related behaviors (Ojala et al., 2012; Rietmeijer-Mentink et al., 2013). Therefore it was assumed that adhering to a healthy nutrition behaviors would be a reactive regulatory strategy, observed more frequently among adolescents with overweight/obesity, than those with normal body weight.

First, it was hypothesized that others’ own weight perceptions (T1) would predict adolescents’ weight status (T2), through *adolescents’ own weight perceptions* (T1) and nutrition behaviors (T1). Second, the association between others’ own weight perceptions (T1) and adolescents’ weight status (T2), mediated by *parental perceptions* (T1) and healthy nutrition (T1) was hypothesized. The third hypothesis was that the association between others’ own weight perceptions (T1) on adolescents’ weight status (T2) would be mediated by *peers’ perceptions* (T1) and healthy nutrition (T2). All hypotheses were tested with weight status at T1 being controlled for. It was hypothesized that the mediated effects of peers’ perceptions and nutrition behaviors would be larger than the mediated effects of parental perceptions and healthy nutrition.

## Materials and Methods

### Participants

At Time 1 (T1), 1103 adolescents (58.1% girls) aged 13 to 20 years old (*M* = 16.62, *SD* = 0.89) with BMIs ranging from 15.52 to 41.21 (*M* = 22.48, *SD* = 4.02) participated in the study. Only 6 (0.5%) adolescents were underweight, 814 (74.5%) had normal body weight, 180 (16.5%) were overweight, and 92 (8.4%) obese. At Time 2 (T2; 13 months later), a total of 614 (56.9% girls) adolescents aged 14 to 20 years old (*M* = 17.19, *SD* = 0.90) with BMIs ranging from 15.64 to 38.93 (*M* = 22.0, *SD* = 3.45) provided their data. At T2, 7 (1.1%) participants were underweight, 466 (76.3%) had normal body weight, 110 (18.0%) were overweight, 28 (4.6%) or obese. Underweight (*n* = 7) was identified as an exclusion criterion in the study, since the mechanisms related to their lower weight might be different than in the groups with normal or excessive weight. The total attrition rate was 44.3%. All participants were white.

Missing data from those who dropped out at T2 were imputed with regression method (maximum likelihood estimation). Therefore, data collected from *N* = 1096 adolescents (58.2% girls) aged 14 to 20 years old (*M* = 17.36, *SD* = 0.85) with BMIs ranging from 16.20 to 41.21 (*M* = 22.38, *SD* = 3.70) were included in the analyses.

### Procedure

The study was conducted in sixteen public middle and high schools in Central and Eastern Poland. All potential respondents lived with their parents (98.9%) or other legal guardians (1.1%) at T1 and T2. Participant and parental consent was obtained prior to the data collection. Individuals were informed about the objectives and the procedure of the study. Those who agreed and provided informed consent were assigned personal codes to secure anonymity and identification across the measurement points. Participants provided their data referring to the perceptions of their body and weight status, nutrition behaviors, perceived parental and peers’ perceptions of their own and adolescents’ weight status, and beliefs about the efficacy of their nutrition and intention to eat healthy. At T1 each participant completed a questionnaire. Afterward, their height and weight were measured privately by researchers in another room or in the school nurse’s office. This procedure was repeated at T2. Researchers were available for consultations after the study completion, if desired. Multiple efforts were made to reduce attrition, and at T2 researchers returned 3–5 times across a three-week period in order to gain access to participants who were willing to respond but were temporarily absent. Attrition was still high, primarily related to finishing, changing or dropping out of school by participants. The study was approved by the Institutional Review Board at the first author’s university.

### Materials

Means, standard deviations, and reliability coefficients are presented in **Table 1.** In line with research conducted so far, the measurement of perceptions of weight status was based on single items (cf. Rudolph et al., 2010; Epuru et al., 2013).

a

**Table 1:** Descriptive statistics, reliability, and correlations between the study variables at T1 and T2 (*N* = 1096).

		*M (SD)*	*α*	2	3	4	5	6	7	8	9
1	T1 Others’ own weight perceptions	5.47 (1.55)	0.41	0.05*	0.12***	0.09**	0.13***	0.06*	0.09***	0.02	-0.01
2	T1 Nutrition behaviors	5.15 (2.06)	0.75		0.10**	0.14***	0.15***	0.06	0.08**	-0.07*	-0.02
3	T1 Adolescents’ own weight perceptions	3.16 (0.82)				0.37***	0.45***	0.26***	0.27***	-0.06*	-0.25***
4	T1 Parental perceptions	2.90 (0.81)					0.53***	0.23***	0.25***	-0.07*	-0.10**
5	T1 Peers’ perception	3.07 (0.79)						0.23***	0.25***	-0.10**	-0.11***
6	T1 Weight status	1.25 (0.43)							0.67***	0.11***	0.07**
7	T2 Weight status	1.25 (0.43)								0.08**	0.08**
8	T1 Age	16.63 (0.89)									-0.01
9	Sex


*^∗∗∗^*p* < 0.001; ^∗∗^*p* < 0.01; ^∗^*p* < 0.05; ^†^*p* < 0.1; T1 = Time 1, baseline; T2 = Time 2, 13-month follow-up; Weight status: 1 = normal, 2 = overweight or obesity.*

#### Others’ Own Weight Perceptions at T1

Others’ own weight perceptions consisted of two items, based on Rudolph et al. (2010). In order to assess it, the respondents were asked “are your parents overweight and trying to lose weight (e.g., eating healthy)” and “are your peers overweight and trying to lose weight (e.g., eating healthy)”. The responses ranged from 1 (definitely not) to 6 (exactly true).

#### Body Weight and Height (T1 and T2)

Biometric measures were assessed with standard medically approved telescopic height measuring rods and floor scales (scale type: BF-100 or BF-25). Age- and gender specific BMI percentiles were calculated with WHO AnthroPlus macro (World Health Organization [WHO], 2007), which is a software package for the global application of the WHO growth reference (de Onis et al., 2007) for children and adolescents. Thus, the BMI indicator of each participant accounts for their age and gender. BMIs were coded onto three weight status categories based on *SD* cut-offs (0 – underweight [less than or equal to 2 *SD*), (1) normal weight, (2) overweight or obesity (≥1 *SD*) based on WHO growth reference (de Onis et al., 2007).

#### Weight Status Perceptions at T1

Three types of weight status perceptions were assessed: (1) adolescents’ own weight perceptions, (2) parental perceptions (parental perceptions of adolescents’ weight status perceived by participants), and (3) peers’ perceptions (peers’ perceptions of adolescents’ weight status perceived by participants). Each construct was measured with one item, based on Epuru et al. (2013). In order to obtain adolescents’ own weight perceptions, the respondents were asked what they think about their weight (“I think I am…”). Adolescents were also asked what their parents (“Looking at me, my parents think I am…”) and peers (“Looking at me, my peers think I am…”) think about their weight status in order to assess parental and peers’ perceptions. The responses in all three items ranged from 1 (very underweight) to 5 (very overweight).

#### Nutrition Behaviors at T1

The definition of healthy nutrition behaviors was provided at the beginning of the questionnaire as balanced, regular meals including fresh fruit and vegetables. In order to evaluate nutrition behaviors, adolescents answered two questions, based on Lally et al. (2011): “How often did you eat a portion of fresh fruit in the last 2 weeks?” and “How often did you eat a portion of vegetables in the last 2 weeks (fresh, boiled, or fried without fat)?”. A portion was defined as the amount that would fit into a cupped hand. The responses were given on a 6-point scale, ranging from 1 (once a week or less) to 6 (four or more times a day).

### Data Analysis

**FIGURE 2:**
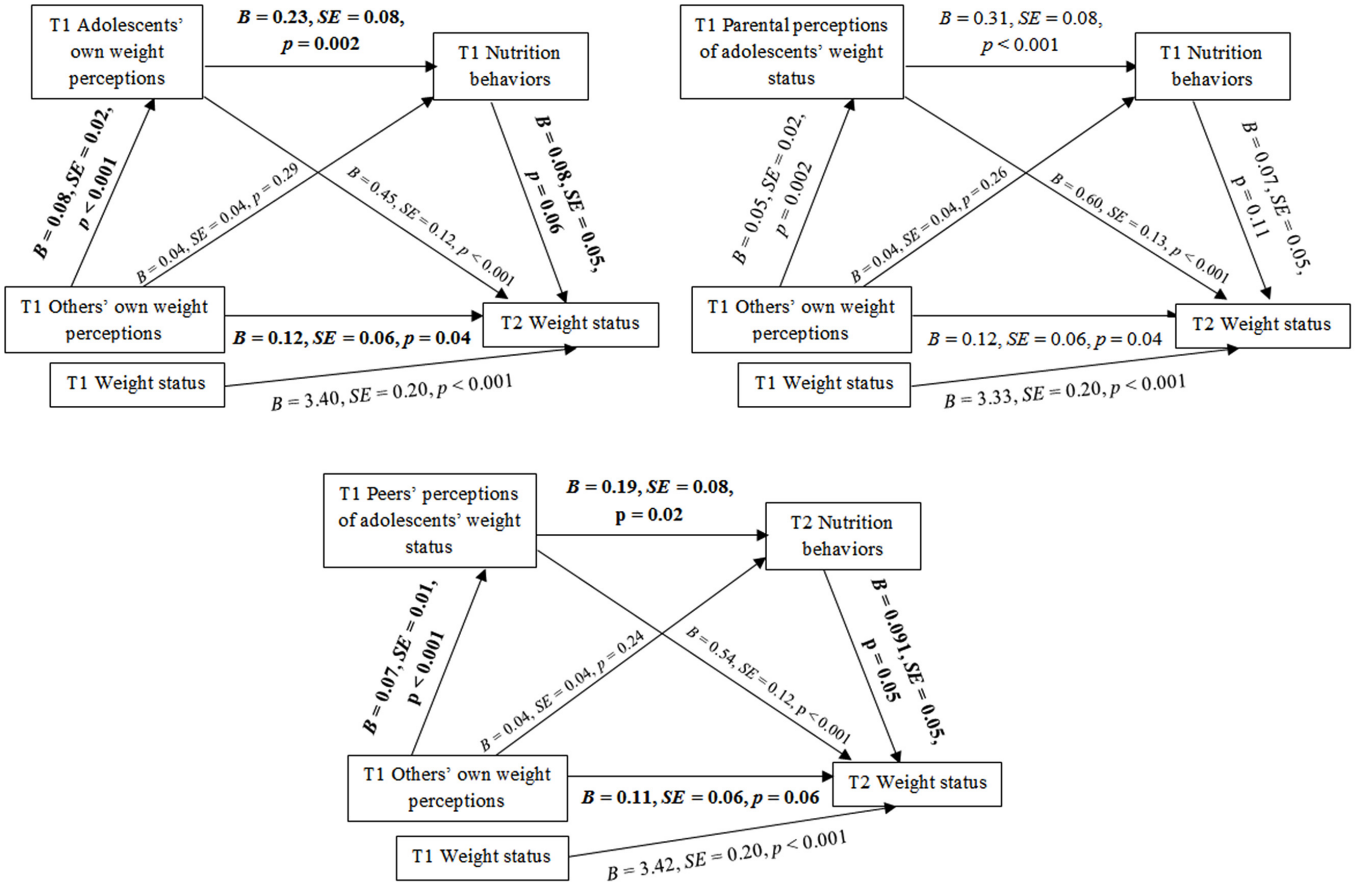
**The mediating effects of weight status perceptions and nutrition behaviors in the relationship between the others’ own weight perceptions and weight status.** T1 = Time 1, baseline; T2 = Time 2, 13-month follow-up; Others’ own weight perceptions = individuals’ perception of what their parents and peers think of their own weight status, Adolescents’ own weight perceptions = what an individual think about their own weight (refers to Model 1), Parental perceptions of adolescents’ weight status = individuals’ perception of what their parents think of the individuals’ weight (refers to Model 2), Peers’ perceptions of adolescents’ weight status = individuals’ perception of what their peers think of the individuals’ weight (refers to Model 3). Significant indirect paths are marked in bold.

Data was analyzed using SPSS version 22. To test whether the relation between others’ own weight perceptions and adolescents’ weight status was mediated by weight status perceptions and nutrition behaviors, we performed three multiple mediation analyses with sequential mediators using PROCESS with 1000 bootstraps (Hayes, 2013). PROCESS permits for conducting multiple mediator regression analysis, accounting for the covariates (T1 weight status). Further, PROCESS allows for testing hypotheses assuming that mediators operate together in a sequence. Results of the analyses are presented using two types of coefficients. A regression coefficient for each parameter is provided (see **Figure 2**). Further, PROCESS estimates the indirect effect coefficient (*B*) for each indirect pathway between the independent variable (IV; T1 others’ own weight perceptions) and the dependent variable (DV; T2 adolescents’ weight status), accounting for respective mediators and covariates (see **Tables 1** and **2**).

**Table 2:** Influence of the others’ own weight perceptions on adolescents’ weight status through the weight status perceptions and nutrition behaviors.

Indirect effects pathways		*B*	*SE*	BC 95% CI	
				
				Lower	Higher
***Hypothesis 1.*** *Testing the influence of the adolescents’ own weight perceptions and nutrition behaviors.*					

Model 1	Others’ own weight perceptions T1 → Adolescents’ own weight perceptions T1 → Weight status T2	**0.03**	0.01	0.02	0.06
	Others’ own weight perceptions T1 → Adolescents’ own weight perceptions T1 → Nutrition behaviors T1 → Weight status T2	**0.002**	0.001	0.0001	0.004
	Others’ own weight perceptions T1 → Nutrition behaviors T1 → Weight status T2	0.003	0.005	–0.002	0.02

***Hypothesis 2.*** *Testing the influence of the parental perceptions and nutrition behaviors.*					

Model 2	Others’ own weight perceptions T1 → Parental perceptions T1 → Weight status T2	**0.03**	0.01	0.01	0.06
	Others’ own weight perceptions T1 → Parental perceptions T1 → Nutrition behaviors T1 → Weight status T2	0.001	0.001	–0.0001	0.004
	Others’ own weight perceptions T1 → Nutrition behaviors T1 → Weight status T2	0.003	0.004	–0.002	0.02

***Hypothesis 3.*** *Testing the influence of the peers’ perceptions and nutrition behaviors.*					

Model 3	Others’ own weight perceptions T1 → Peers’ perceptions T1 → Weight status T2	**0.04**	0.01	0.02	0.06
	Others’ own weight perceptions T1 → Peers’ perceptions T1 → Nutrition behaviors T1 → Weight status T2	**0.001**	0.001	0.0001	0.004
	Others’ own weight perceptions T1 → Nutrition behaviors T1 → Weight status T2	0.004	0.005	–0.001	0.02


*Values of indirect effect coefficient (*B*) presented in bold are significant. Each bootstrap was based on 1,000 repetitions. Bias corrected (BC) confidence intervals (CI) that do not include zero indicate a significant indirect effect. T1 = Time 1, baseline; T2 = Time 2, 13-month follow-up. Significant coefficients are marked in bold.*

In the current study the IV was others’ own weight perceptions (T1); the DV in all analyses was adolescents’ objectively measured weight status (T2); the first mediators were three types of weight status perceptions (T1) and the second mediators were adolescents’ nutrition behaviors (T1).

Longitudinal research using multiple mediation analysis with sequential mediators was conducted to account for long-term effects of adolescents’ weight perceptions and nutrition behaviors on changes in adolescents’ weight status (Cislak et al., 2012). The first mediators in respective models were participants’ own weight perceptions, or parental perceptions, or peers’ perceptions measured at T1 and the second mediator in all models was adolescents’ nutrition behaviors measured at T1. Further, weight status at T1 was controlled for.

As suggested by MacKinnon (2008), the IV, the mediators, and the DV in the respective equations were measured at different time points (T1 and T2) in order to establish temporal precedence. Missing data were imputed with multiple imputation, which is an effective way of treating data, even if up to 50% of it is missing (Pigott, 2001). A dataset created by averaging five data sets with imputed values was used in analyses. The attrition analysis is presented below.

### Ethical Statement

All procedures performed in studies involving human participants were in accordance with the ethical standards of the Institutional Review Board and Ethics Committee at University of Social Sciences and Humanities, Poland. Informed consent was obtained from all adolescents participating in the study and from their parents (in case adolescents were younger than 18 years old).

## Results

### Preliminary Analysis

#### Attrition Analysis

Completers did not differ from those who dropped out at T2 in terms of any of the indicators of weight status perceptions, others’ own weight perceptions, nutrition behaviors, and weight status, all *F*s < 2.76, *p*s > 0.10, or sex, χ_(1)_^2^ = 0.11, *p* = 0.75. Dropouts and completers differed in terms of age, *F*(1,1102) = 63.07, *p* < 0.001 with dropouts being slightly older (*M* = 16.87, *SD* = 0.69) than completers (*M* = 16.43, *SD* = 0.98, Cohen’s *d* = 0.51 [95% CI: 0.46 to 0.56]).

#### Results of Correlation Analyses and Weight Status Changes

Correlations between study variables for the total sample (*N* = 1096) are presented in **Table 1.** There were weak positive correlations between nutrition behaviors (T1) and weight status perceptions (T1). Moderate positive associations were found between all three types of weight status perceptions (T1). Moreover, higher levels of weight status perceptions (T1) were moderately, but significantly correlated with higher adolescents’ weight status (T1 and T2).

There were some changes in weight status from T1 to T2, in particular there were fewer adolescents with overweight/obesity and more with normal weight status at T2, χ_(4)_^2^ = 559.99, *p* < 0.001, η^2^ = 0.02 (T1 weight status: normal weight 74.9%, overweight or obesity 25.1%; T2 weight status: normal weight 75.1%, overweight or obesity 24.9%). However, considering mean BMI values, the effects were small and it may be considered that they do not differ significantly from zero (Cohen’s *d* = 0.09 [95% CI: –0.09 to 0.27]).

### Testing the Mediating Effect of the Adolescents’ Own Weight Perceptions

Model 1 was designed to verify the mediating effects of multiple mediators: *adolescents’ own weight perceptions* (T1) and nutrition behaviors (T1) in the relationship between others’ own weight perceptions (T1) (IV) and weight status (T2) (DV). Hypothesized associations between others’ own weight perceptions and adolescents’ weight status are presented in **Figure 2.** Indirect effects of others’ own weight perceptions (T1) on weight status (T2) through adolescents’ own weight perceptions (T2) and nutrition behaviors (T2) was tested with weight status (T1) being controlled for.

The results of mediation analysis for Model 1 (**Table 2**) showed that the association between others’ own weight perceptions (T1) and weight status (T2) was mediated by *adolescents’ own weight perceptions* (T1) and by the multiple mediators: *adolescents’ own weight perceptions* (T1) and nutrition behaviors (T1) as indicated by the significant indirect effect. Direct association between IV (T1), mediators (T1), and DV (T2) are presented in **Figure 2.**

### Testing the Mediating Effect of the Parental Perceptions

Model 2 verified the mediating effects of multiple mediators: *parental perceptions* (T1) and nutrition behaviors (T1) in the relationship between others’ own weight perceptions (T1) (IV) and weight status (T2) (DV) (see **Figure 2**). Indirect effects of others’ own weight perceptions (T1) on adolescents’ weight status (T2) through parental perceptions (T1) and nutrition behaviors (T1) was tested controlling for weight status at T1.

The results of analysis conducted for Model 2 (**Table 2**) indicated no significant indirect effects of *parental perceptions* (T1) and nutrition behaviors (T1) in the relationship between IV (T1) and weight status (T2). Direct association between IV (T1), mediators (T1), and DV (T2) are presented in the **Figure 2.**

### Testing the Mediating Effect of the Peers’ Perceptions

Model 3 tested the mediating effects of multiple mediators: *peers’ perception* (T1) and nutrition behaviors (T1) in the relationship between others’ own weight perceptions (T1) (IV) and weight status (T2) (DV) (see **Figure 2**). Indirect effects of others’ own weight perceptions (T1) on adolescents’ weight status (T2) through peers’ perceptions (T2) and nutrition behaviors (T2) was tested controlling for weight status at T1.

The results of mediation analysis for Model 3 (**Table 2**) showed that the association between others’ own weight perceptions (T1) and weight status (T2) was mediated by *peers’ perceptions* (T1), and by the multiple mediators: *peers’ perceptions* (T1) and nutrition behaviors (T12) as indicated by the significant indirect effect. Direct association between IV (T1), mediators (T1), and DV (T2) are presented in the **Figure 2.**

## Discussion

This longitudinal study provides novel evidence for the associations between adolescents’ subjective cognitions of others’ own weight perceptions, weight status perceptions (including adolescents’ own weight perceptions, parental perceptions, and peers’ perceptions), adolescents’ nutrition behaviors, and their weight status. Changes in weight status among adolescents with normal and excessive weight were followed up for 13 months.

The results confirm the indirect effects of others’ own weight perceptions on adolescents’ weight status through *adolescents’ own weight perceptions* and nutrition behaviors, and *peers’ perceptions* and nutrition behaviors. Higher levels of others’ own weight perceptions (T1) were associated with higher levels of *adolescents’ own weight perceptions* (T1) or *peers’ perceptions* (T1), higher intake of healthy foods (T1), and adolescents more likely to be overweight/obese at T2. On the other hand, no indirect effects of others’ own weight perceptions on adolescents’ weight status through *parental perceptions* and nutrition behaviors were found. Thus, adolescents’ own weight perceptions (T1) and nutrition behaviors (T1), and peers’ perceptions (T1) and nutrition behaviors (T1) act as significant mediators of the relationship between the others’ own weight perceptions (T1) and adolescents’ weight status (T2).

Our findings suggest that others’ own weight perceptions are associated with healthier nutrition behaviors but also higher likelihood of adolescents perceiving themselves as overweight and higher weight status. Similar results have been found in previous research (Almenara et al., 2014) with male adolescents, indicating that being overweight or obese, or just perceiving oneself to be so is connected with higher reports of healthy nutrition. Similarly, Khambalia et al. (2012) indicated that adolescents who were overweight or perceived themselves to be so had higher odds of trying to lose weight (being physically active or/and eat healthy). Our findings are also congruent with the results obtained by Race Mackey and La Greca (2008), who indicated that adolescent girls’ perceptions of their own weight status predicted their weight control behaviors. Chen et al. (2014) also found that weight perceptions status in the group of healthy and excessive weight adolescents were associated with more self-reported attempts to lose weight. These cross-sectional studies, however, only explored bivariate associations of cognitions and nutrition behaviors. Our data go beyond this research, as we demonstrated longitudinally that adolescents’ own weight perceptions and their nutrition behaviors mediate the relationship between others’ own weight perceptions and adolescents’ weight status.

The results of this study indicate no mediating effects of parental perceptions and nutrition behaviors in the association between others’ own weight perceptions and adolescents’ weight status, although other research has found at least bivariate connections between these factors (Eckstein et al., 2006; Tompkins et al., 2014). However, Teun et al. (2014) also showed no associations between parental perceptions of adolescents’ obesity and adolescents’ weight-control behaviors or parental intentions to improve their children’s health behaviors, which is congruent with our findings. Similarly, Chen et al. (2014) found no significant connections between parental perceptions of adolescents’ weight status and adolescents’ attempts to lose weight. Previous studies did not take into account parental perceptions of adolescents’ weight as perceived by these adolescents, though. Thus, our research provides new evidence that among the three types of overweight status perceptions (i.e.. adolescents’ own weight perceptions, parental perceptions, and peers’ perceptions), parental perceptions play the weakest role in prediction of nutrition behaviors during adolescence.

Furthermore, our results demonstrate that others’ own weight perceptions are associated with higher levels of peers’ perceptions, their nutrition behaviors and weight status. To our knowledge, there are few studies that have explored the associations between the peers’ perceptions of adolescents’ weight and adolescents’ weight or nutrition behaviors, as the research has mainly focused on peer weight norms perceptions (Hutchinson and Rapee, 2007; Race Mackey and La Greca, 2008; Perkins et al., 2014). Despite this fact, similar variables (self, parents and peers’ perceptions about weight status) have been used and similar findings have been obtained in a cross-sectional study conducted by Epuru et al. (2013), showing that weight perceptions are not a good indicator for weight management practices. However, this research has focused mainly on self-perceptions. Therefore, our data provides novel evidence indicating not only direct effects of the peers’ perceptions, but also their indirect effects (sequential with adolescents’ nutrition behaviors) in association with others’ own weight perceptions and adolescents’ weight status (measured objectively). Moreover, peers’ perceptions as well as adolescents’ own weight perceptions are found to be better predictors of adolescents’ nutrition behaviors than parental perceptions.

The main limitation of the study is the high attrition rate. The results may also be affected by the exclusion of underweight participants. However, this was chosen as the group at the highest risk of being overweight or obese and expected to experience health consequences in the future. The measurement of weight-related perceptions used one or two items per construct, which is in line with approach used in previous research (Rudolph et al., 2010; Epuru et al., 2013). Such measures, however, have limited reliability. Another limitation might be not taking into account emotional and self-regulatory factors that were found to be important aspects in the development and maintenance of obesity (e.g., emotion regulation; Raman et al., 2013). Future research could include these variables and investigate their synergistic effects in order to provide the best explanation of adolescents’ nutrition behaviors and unfavorable body weight changes. Moreover, future research could include other cognitive and social influence variables which predict BMI changes in adolescents (e.g., weight discrepancies, parental modeling; Zarychta et al., 2014, 2016). In addition to healthy nutrition behaviors, adolescents with excessive weight may engage in restrictive eating which have distinct effects on adolescents’ weight (Neumark-Sztainer et al., 2012). Future research should consider the interplay between healthy nutrition behavior (e.g., maintaining diet rich in fruit and vegetable) and restrictive eating as the mediators between adolescents’ weight perceptions and their BMI changes.

## Conclusion

The great majority of previous research has investigated direct effects of others’ own weight perceptions on adolescents’ weight status or weight-related behaviors (Hisar and Toruner, 2013; Chen et al., 2014; Perkins et al., 2014). However, the existing evidence did not explore the interplay between different types of weight status perceptions (i.e., adolescents’ own weight perceptions, others’ own weight, and adolescents’ weight perceptions) or their synergistic effects on weight status and behavior. Our research was novel in the way of treating weight status perceptions and nutrition behaviors as sequential mediators in the relationship between the others’ own weight perceptions and adolescents’ weight status. Moreover, we verify the subjective perception of tested variables in association in objectively measured weight status, what was previously found to be of higher significance in prediction of the healthy behavior than objective recognition of actual weight (Perkins et al., 2014). To our knowledge, this is the first study to show these three types of weight status perceptions have distinct effects in relations with adolescents’ nutrition behaviors, their weight status and the others’ own weight perceptions. The previous research did not investigate which of these perceptions is stronger or weaker predictor of adolescents’ nutrition behaviors. Our research fills that gap indicating the parental perceptions as being the weakest and insignificant one. Only *the adolescents’ own weight perceptions* and *the peers’ perceptions* were found to be significant mediators acting sequential with nutrition behaviors. It means that during the adolescence it is important what the participants think about their own weight status and what they think of their peers’ perceptions of adolescents’ weight status, and not what they think of their parents’ perceptions of adolescents’ weight status in case of effectively helping adolescents to engage in healthy nutrition behaviors. Thus, our hypothesis that the mediating effects of the peers’ perceptions and nutrition behaviors would be larger than the mediating effects of the parental perceptions and nutrition behaviors have been proven right.

Our research is particularly important as BMI and healthy nutrition behaviors in adolescents are difficult to change (Young et al., 2004; Robertson et al., 2014). Excessive weight in adolescence may have long term health-related consequences (World Health Organization [WHO], 2012). Therefore, modifiable determinants of these behaviors have relevant implications on overweight prevention and treatment. Modification of a range of weight-related perceptions of adolescents may be easily incorporated into any psychosocial program facilitating weight loss. Obesity prevention programs may also educate adolescents about the impact that their weight-related perceptions have on nutrition behaviors and weight status. Adolescents may learn to identify different types of weight-related perceptions and to modify them. Modifications of a range of weight-related perceptions would enable adolescents to influence their nutrition behaviors and, in turn, their BMI in the most beneficial way.

Even though there were less adolescents with overweight/obesity at T2 (small and insignificant change in weight status), the results of this study indicate that healthy nutrition was connected with higher weight status measured at T2. It is essential to emphasize that overweight/obese adolescents in our research made attempts to change their weight status through healthier nutrition behaviors. These findings indicate that the weight status perceptions and nutrition behaviors are most likely to be reactive to an increased weight status. Further, not only the role of others’ own weight perceptions, but also the role of two out of three types of overweight status perceptions (i.e., the adolescents’ own weight perceptions and the peers’ perceptions) should be taken into account when considering adolescents’ overweight and obesity prevention and treatment programs, as they were found to be significant in terms of adolescents’ healthy nutrition behaviors.

## Author Contributions

KZ conceived of the study, participated in its design, performed the statistical analysis, drafted and revised the manuscript; BM participated in the design and interpretation of the data and helped to draft the manuscript; AL conceived of the study, participated in its design, and coordination, participated in the interpretation of the data and helped to draft the manuscript. All authors read and approved the final manuscript.

## Conflict of Interest Statement

The authors declare that the research was conducted in the absence of any commercial or financial relationships that could be construed as a potential conflict of interest.
